# Development, validation, and visualization of a machine learning-based predictive model for sarcopenia risk in patients with diabetes mellitus

**DOI:** 10.3389/fmed.2026.1904187

**Published:** 2026-08-14

**Authors:** Mingjie Tang, Shiyi Zheng, Hang Xu, Yinghong Li, Chaoming Chen

**Affiliations:** 1 Nanjing Hospital of Chinese Medicine Affiliated to Nanjing University of Chinese Medicine, Nanjing, China; 2 Nanjing University of Chinese Medicine, Nanjing, China

**Keywords:** diabetes mellitus, machine learning, predictive model, sarcopenia, SHAP

## Abstract

**Background:**

Sarcopenia is a common complication in patients with diabetes mellitus (DM), yet early screening remains challenging due to the lack of accessible tools. This study aimed to develop and externally validate a machine learning (ML) -based predictive model for sarcopenia risk in DM patients, with enhanced interpretability.

**Methods:**

Data from 1,074 DM patients at a single center (training and internal validation) and two external datasets (NHANES, *n* = 1,078; CHARLS, *n* = 547) were used. Seven ML methods were evaluated. Feature selection used LASSO and Boruta. Model performance was assessed by AUC, calibration (Brier score), and decision curve analysis. SHAP was applied to explain the final model.

**Results:**

Six predictors (age, sex, BMI, hemoglobin, uric acid, creatinine) were identified. In internal validation, LightGBM achieved the highest AUC (0.973). However, on both external datasets, logistic regression showed superior and stable performance (NHANES AUC = 0.967, CHARLS AUC = 0.949), with lower Brier scores (0.042 and 0.061) and favorable net benefit. LightGBM’s performance decreased externally (AUC 0.873 and 0.868). Therefore, logistic regression was selected as the final model. SHAP analysis revealed that low BMI, older age, and low creatinine were the strongest predictors of sarcopenia.

**Conclusion:**

Using six readily available clinical features, the logistic regression model shows robust generalizability across populations. This clinically practical tool supports early risk identification in diabetic patients, enabling timely interventions to mitigate sarcopenia-related complications.

## 1 Introduction

Diabetes mellitus (DM) has emerged as a major chronic metabolic disease posing a substantial threat to global public health (1, 2). According to the 2024 International Diabetes Federation (IDF) Atlas, the global diabetic population has reached 589 million, with projections indicating a rise to 853 million by 2050 (3). Sarcopenia, a geriatric syndrome characterized by progressive loss of skeletal muscle mass and function, demonstrates a markedly higher prevalence among diabetic patients compared with the general population, with reported rates ranging from 7 to 29.3% (4, 5). With the rapid aging of the population and extended disease course of diabetes, the concurrent presentation of sarcopenia and diabetes is rising sharply, posing a substantial and escalating threat to global public health (6, 7).

A complex bidirectional pathophysiological interplay exists between sarcopenia and diabetes (8). Shared mechanisms including insulin resistance, chronic low-grade inflammation, oxidative stress, and mitochondrial dysfunction not only accelerate muscle protein degradation while inhibiting synthesis but also increase the risk of falls, fractures, and significantly diminished quality of life (9–11). Diabetic patients with concurrent sarcopenia exhibit elevated risks of hospitalization, surgical complications, and all-cause mortality, alongside a substantially increased 10-year atherosclerotic cardiovascular disease (ASCVD) risk (12–16). Nevertheless, sarcopenia diagnosis remains heavily dependent on specialized equipment such as dual-energy X-ray absorptiometry (DXA) or bioelectrical impedance analysis (BIA) (17), which is difficult to implement in routine clinical practice, especially in primary care and community settings. Therefore, a novel predictive tool is urgently needed for early and precise screening of sarcopenia in patients with diabetes.

Advances in artificial intelligence (AI) and machine learning (ML) have enabled high-dimensional analysis and identification of risk factor interactions, with increasing application in clinical risk stratification (18, 19). In a systematic review encompassing 155 AI-driven diabetes prediction studies, ensemble methods including Random Forest and XGBoost achieved substantially higher accuracy (85–90%) than conventional approaches (60–75%), demonstrating a consistent performance advantage for ML-based models (20). Furthermore, SHapley Additive exPlanations (SHAP) addresses the “black box” nature of ML by offering model interpretability (21). Two previous studies developed ML-based predictive models for sarcopenia in patients with DM, but both lacked interpretability and external validation (22, 23).

Therefore, this study aims to develop and validate a machine learning-based predictive model for sarcopenia risk in patients with diabetes mellitus using data from a single-center retrospective cross-sectional study, with external validation performed using two public external databases. Furthermore, this study employs SHAP to quantify feature importance and interpret the model’s prediction outputs, to enhance model interpretability.

## 2 Materials and methods

### 2.1 Study design and data source

**Figure 1:**
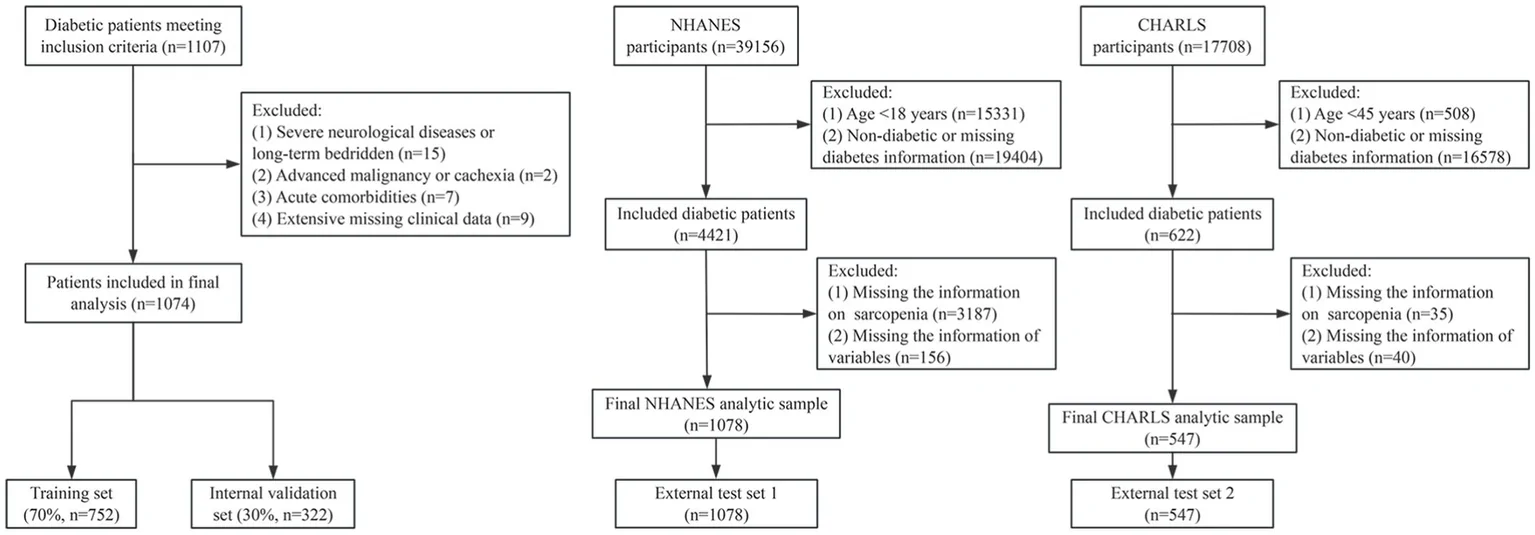
Study flow diagram.

This study adopted a cross-sectional design. Data were collected from patients with DM admitted to Nanjing Hospital of Chinese Medicine between March 2024 and March 2026. Inclusion criteria were as follows: (1) age between 18 and 90 years; (2) confirmed diagnosis of DM; (3) ability to independently complete sarcopenia assessments. A total of 1,107 patients met the inclusion criteria. Exclusion criteria included: (1) concurrent severe neurological diseases or long-term bedridden status (*n* = 15); (2) advanced malignancy or cachexia (*n* = 2); (3) acute comorbidities such as acute infection, acute pulmonary embolism, or acute myocardial infarction (*n* = 7); (4) extensive missing clinical data (*n* = 9). Finally, data from 1,074 patients were included in the analysis (Figure 1). The final analysis included 674 male (62.76%) and 400 female (37.24%) participants. This study was approved by the Ethics Committee of Nanjing Hospital of Chinese Medicine (No.2026KY094), and was adhered to the Declaration of Helsinki. Due to the retrospective nature of the study and anonymized data, the requirement for patient informed consent was waived.

Two external test datasets were also used, namely the National Health and Nutrition Examination Survey (NHANES) and the China Health and Retirement Longitudinal Study (CHARLS). The NHANES program aims to evaluate the nutritional and general health status of the American population (24). NHANES data from 2011 to 2018 were used, including 1,078 participants. The NHANES protocol was approved by the National Center for Health Statistics Institutional Review Board. The CHARLS initiative was designed to collect comprehensive, high-quality data from elderly households and individuals in China. CHARLS baseline data from 2011 were used, including 547 participants. The CHARLS study received approval from the Biomedical Ethics Review Committee of Peking University (IRB00001052-11015) (25). Detailed screening procedures are shown in Figure 1.

### 2.2 Participants

This study included patients with DM who met at least one of the following diagnostic criteria: (1) previous diagnosis of DM by a physician; (2) fasting plasma glucose (FPG) ≥ 7 mmol/L; (3) glycated hemoglobin (HbA1c) ≥ 6.5%.

### 2.3 Outcomes

In hospital and CHARLS datasets, sarcopenia was defined following the 2025 Asian Working Group for Sarcopenia (AWGS) criteria (26) as low muscle mass combined with low muscle strength. For low muscle mass assessment, the hospital dataset used BIA (InBody770) to directly measure appendicular skeletal muscle mass and calculate appendicular skeletal muscle mass (ASM)/height^2^. The CHARLS dataset estimated ASM using a formula that has been validated in multiple studies (27, 28): ASM (kg) = 0.193 × body weight (kg) + 0.107 × height (cm) − 4.157 × sex (male = 1, female = 2) − 0.037 × age (years) − 2.631, followed by calculation of ASM/height^2^. Both datasets applied the AWGS 2025 age-stratified thresholds for low muscle mass: for individuals aged <65 years, <7.6 kg/m^2^ for men and <5.7 kg/m^2^ for women; for those aged ≥65 years, <7.0 kg/m^2^ for men and <5.7 kg/m^2^ for women. Low muscle strength was defined with age-specific cutoffs: handgrip strength <34 kg for men and <20 kg for women for those aged <65 years, and ≤28 kg for men and ≤18 kg for women for those aged ≥65 years. In NHANES dataset, muscle mass was measured using DXA. According to the European Working Group on Sarcopenia in Older People 2 (EWGSOP2) criteria (17), sarcopenia was defined by thresholds of ASM/height^2^ < 7.0 kg/m^2^ for men and <5.4 kg/m^2^ for women.

### 2.4 Data collection and preprocessing

Variable selection was guided by knowledge established in previous studies (22, 23) and included four parts. Part 1 collected basic patient information including age and sex. Part 2 included behavioral factors: smoking and alcohol consumption. Part 3 included history of common chronic diseases: hypertension, cardiovascular disease (CVD), and stroke. Part 3 also included routine blood and biochemical indicators: white blood cell count (WBC), neutrophils (NEU), lymphocytes (LYM), monocytes (MONO), red blood cell count (RBC), hemoglobin (Hb), mean corpuscular volume (MCV), mean corpuscular hemoglobin (MCH), mean corpuscular hemoglobin concentration (MCHC), red cell distribution width (RDW), platelet count (PLT), platelet distribution width (PDW), total cholesterol (TC), triglycerides (TG), high density lipoprotein cholesterol (HDL-C), low density lipoprotein cholesterol (LDL-C), fasting blood glucose (FBG), creatinine, uric acid, and glycated hemoglobin (HbA1c). Part 4 included body mass index (BMI) and waist circumference (WC).

The missing data pattern in the original hospital dataset was examined using Little’s test for missing completely at random (MCAR), which yielded a statistically significant result (χ^2^ = 189.16, *p* < 0.01), indicating that the data were not MCAR (29). The proportion of missing values for each variable was uniformly low, all below 5%. To evaluate the potential impact of the non-MCAR mechanism and the subsequent imputation strategy, we performed a sensitivity analysis by comparing model performance between the dataset after multiple imputation and the dataset with complete cases only, using the DeLong test for AUC comparison. The results demonstrated no significant difference in discriminative performance between the two approaches (Supplementary Table S1), supporting the robustness of the imputation procedure and the subsequent analyses. Therefore, we applied multiple imputation by chained equations (MICE) to handle missing values. The distribution of missing data is visualized in Supplementary Figure S1.

### 2.5 Sample size estimation

This study followed sample size estimation principles for prognostic model development. Given the sarcopenia prevalence of 17.3% in the hospital dataset, based on the events per variable (EPV) requirement of at least 10 events per predictor (30), we planned to include no more than 10 candidate predictors in the logistic regression model, requiring at least 100 positive cases. A total of 186 positive cases were finally collected, which largely met the event number requirement for model stability. The final model included 6 predictors, well below the maximum of 18 permitted by the EPV principle (186/10 = 18.6).

### 2.6 Selection of variables

The variable selection procedure was conducted as follows. First, LASSO regression and the Boruta algorithm were independently applied to screen the 29 variables in the training set, and the intersection of the variables selected by both methods was taken. LASSO regression used 10-fold cross validation for data processing and variable selection, identifying predictors based on features with nonzero coefficients (31). The Boruta algorithm, integrated with random forests through multiple iterations, performed feature selection to avoid overfitting and ensure the stability of the selected features (32). Subsequently, multicollinearity diagnostics were performed on these intersected variables by calculating the variance inflation factor (VIF) (33), and variables with VIF > 10 were excluded (Supplementary Table S2). Finally, the remaining variables were entered into multivariable logistic regression analysis, with a threshold of *p* < 0.05 considered statistically significant.

### 2.7 Model construction, evaluation, and testing

The model development and validation procedure was conducted as follows. First, the hospital dataset was randomly divided into a training set and a test set at a ratio of 7:3, and 5-fold cross validation was employed to optimize performance and prevent overfitting. Second, seven machine learning algorithms were evaluated: logistic regression, decision tree, random forest, XGBoost, LightGBM, support vector machine (SVM), and artificial neural network (ANN). Third, two external validation datasets were used to assess the generalizability of the model.

Model performance was evaluated using accuracy, sensitivity, specificity, precision, F1 score, brier score, and the area under the receiver operating characteristic curve (AUC) (34). An AUC value closer to 1.0 indicates stronger discriminative ability, whereas a value near 0.5 suggests predictions close to random. DeLong test was used to determine whether there were significant differences in AUC values among different models. Calibration curves were used to quantify agreement between predicted and observed outcomes (35), and decision curve analysis (DCA) was used to evaluate clinical net benefit (36). Finally, SHAP analysis was performed using the optimal model to calculate the contribution of each feature to the prediction outcome, providing both local and global explanations (37, 38).

### 2.8 Statistical analysis

Categorical variables were expressed as frequency (percentage) and analyzed using the chi square test. Normally distributed continuous variables were presented as mean ± standard deviation (SD) and compared using one way analysis of variance (ANOVA), while non normally distributed variables were summarized as median (interquartile range, IQR) and analyzed using the Kruskal Wallis test. A two sided *p* < 0.05 was considered statistically significant. All analyses were performed using R 4.5.2.

## 3 Results

### 3.1 Baseline characteristics

A total of 1,074 patients with DM were included, with 186 (17.3%) in the sarcopenia group and 888 (82.7%) in the non-sarcopenia group. Baseline characteristics are shown in Table 1. Compared with the non-sarcopenia group, the sarcopenia group was older (*p* < 0.001), had a higher proportion of females (*p* = 0.001), and a higher prevalence of CVD (*p* = 0.006). Levels of Hb, PLT, TG, creatinine, uric acid, BMI, and WC were significantly lower in the sarcopenia group (*p* < 0.05), whereas HDL-C and HbA1c were significantly higher (*p* < 0.05).

**Table 1:** Comparison of clinical data between participants with or without sarcopenia.

Variable	Overall (*n* = 1,074)	No sarcopenia (*n* = 888)	Sarcopenia (*n* = 186)	*P*
Age, Median (IQR)	61.00 (50.00, 69.00)	59.00 (47.00, 68.00)	68.00 (61.00, 76.00)	<0.001
Sex, *n* (%)				0.001
Male	674 (62.76)	578 (65.09)	96 (51.61)	
Female	400 (37.24)	310 (34.91)	90 (48.39)	
Hypertension, *n* (%)				0.056
No	564 (52.51)	454 (51.13)	110 (59.14)	
Yes	510 (47.49)	434 (48.87)	76 (40.86)	
CVD, *n* (%)				0.006
No	954 (88.83)	800 (90.09)	154 (82.80)	
Yes	120 (11.17)	88 (9.91)	32 (17.20)	
Stroke, *n* (%)				0.899
No	848 (78.96)	700 (78.83)	148 (79.57)	
Yes	226 (21.04)	188 (21.17)	38 (20.43)	
Smoking, *n* (%)				0.580
No	788 (73.37)	648 (72.97)	140 (75.27)	
Yes	286 (26.63)	240 (27.03)	46 (24.73)	
Alcohol, *n* (%)				
No	890 (82.87)	734 (82.66)	156 (83.87)	0.770
Yes	184 (17.13)	154 (17.34)	30 (16.13)	
WBC, Median (IQR)	6.39 (5.44, 7.56)	6.46 (5.49, 7.57)	6.21 (5.29, 7.41)	0.177
NEU, Median (IQR)	3.78 (3.00, 4.68)	3.82 (3.00, 4.72)	3.63 (2.97, 4.37)	0.146
LYM, Median (IQR)	1.95 (1.56, 2.36)	1.95 (1.58, 2.38)	1.88 (1.50, 2.29)	0.094
MONO, Median (IQR)	0.39 (0.32, 0.48)	0.39 (0.32, 0.47)	0.40 (0.31, 0.49)	0.377
RBC, Median (IQR)	4.57 (4.23, 4.93)	4.58 (4.24, 4.94)	4.51 (4.20, 4.88)	0.274
Hb, Median (IQR)	138.00 (128.00, 149.00)	139.00 (129.00, 151.00)	133.00 (120.00, 142.00)	<0.001
MCV, Median (IQR)	90.50 (87.70, 93.47)	90.50 (87.70, 93.60)	90.40 (87.80, 92.70)	0.555
MCH, Median (IQR)	30.10 (29.20, 31.20)	30.10 (29.20, 31.20)	30.10 (29.30, 31.10)	0.811
MCHC, Median (IQR)	333.00 (329.00, 337.00)	333.00 (329.00, 337.00)	333.00 (328.00, 339.00)	0.455
RDW, Median (IQR)	13.00 (12.60, 13.40)	13.00 (12.60, 13.40)	13.00 (12.70, 13.50)	0.098
PLT, Median (IQR)	191.00 (160.00, 230.00)	193.00 (162.00, 232.25)	184.00 (155.00, 223.00)	0.016
PDW, Median (IQR)	16.40 (16.10, 16.60)	16.40 (16.10, 16.60)	16.40 (16.10, 16.60)	0.872
TC, Median (IQR)	4.59 (3.76, 5.47)	4.60 (3.71, 5.46)	4.56 (3.98, 5.54)	0.994
TG, Median (IQR)	1.31 (0.90, 2.20)	1.38 (0.94, 2.37)	1.15 (0.79, 1.52)	<0.001
HDL. C, Median (IQR)	1.15 (0.99, 1.37)	1.13 (0.97, 1.32)	1.28 (1.08, 1.48)	<0.001
LDL. C, Median (IQR)	2.53 (1.95, 3.20)	2.55 (1.95, 3.19)	2.46 (1.95, 3.36)	0.964
FBG, Median (IQR)	7.44 (5.36, 11.07)	7.44 (5.42, 11.14)	7.32 (5.23, 10.04)	0.422
Creatinine, Median (IQR)	63.00 (52.00, 76.00)	65.00 (54.00, 77.93)	55.00 (46.50, 68.30)	<0.001
Uric Acid, Median (IQR)	311.60 (256.00, 387.00)	316.95 (264.00, 391.00)	271.00 (225.00, 350.00)	<0.001
HbA1c, Median (IQR)	8.59 (7.01, 10.50)	8.50 (7.00, 10.40)	8.84 (7.82, 10.72)	0.021
BMI, Median (IQR)	24.49 (22.24, 26.95)	25.16 (23.14, 27.66)	21.48 (20.33, 23.03)	<0.001
WC, Median (IQR)	86.50 (80.50, 95.20)	89.10 (82.60, 96.73)	79.60 (74.60, 83.80)	<0.001

Compared with the non-sarcopenia group, the sarcopenia group was significantly older, had a higher proportion of females and CVD, and exhibited lower levels of Hb, PLT, TG, creatinine, uric acid, BMI, and WC, while HDL-C and HbA1c were significantly higher (all *p* < 0.05). BMI, Body mass index; CVD, Cardiovascular disease; FBG, Fasting blood glucose; Hb, Hemoglobin; HbA1c, Glycated hemoglobin; HDL-C, High-density lipoprotein cholesterol; IQR, Interquartile range; LDL-C, Low-density lipoprotein cholesterol; LYM, Lymphocyte count; MCH, Mean corpuscular hemoglobin; MCHC, Mean corpuscular hemoglobin concentration; MCV, Mean corpuscular volume; MONO, Monocyte count; NEU, Neutrophil count; PDW, Platelet distribution width; PLT, Platelet count; RBC, Red blood cell count; RDW, Red cell distribution width; TC, Total cholesterol; TG, Triglycerides; WBC, White blood cell count; WC, Waist circumference.

### 3.2 Feature selection

**Figure 2:**
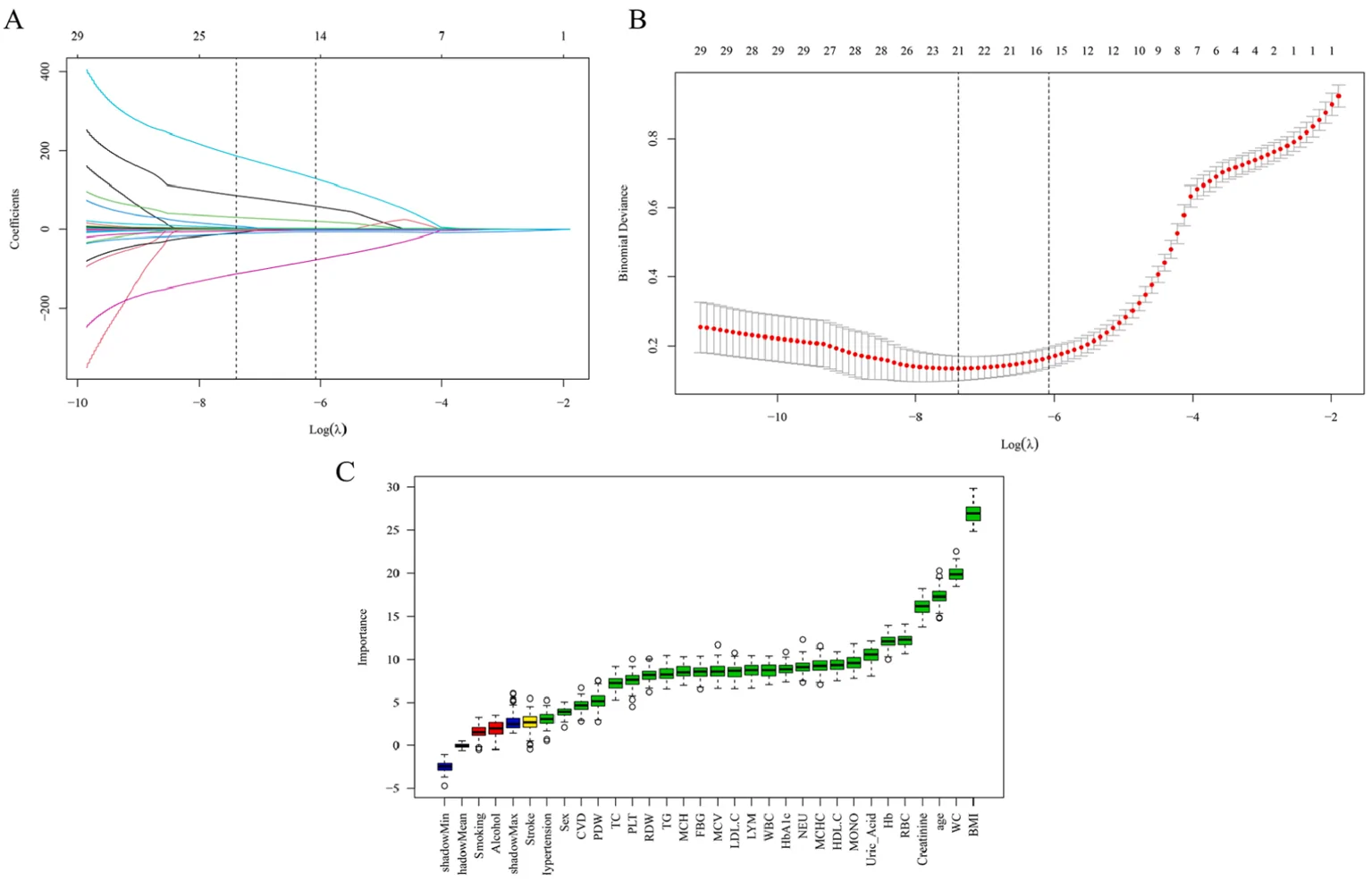
Variable selection using LASSO regression and the Boruta algorithm. **(A)** LASSO cross-validation for *λ* selection. Dashed lines indicate *λ_min* and *λ_1se*. **(B)** LASSO coefficient paths. **(C)** Boruta algorithm output with variable importance.

Based on clinical experience and literature, 29 candidate variables were initially included. LASSO regression was performed with 10-fold cross validation and the 1 standard error criterion (*λ*₁-SE) to select variables with non-zero coefficients; the Boruta algorithm was applied independently (Figure 2). The intersection of variables selected by both methods yielded 13 variables. Multicollinearity diagnostics were then conducted, excluding variables with VIF greater than 10 (MCV and MCHC). The remaining variables were entered into multivariable logistic regression analysis, ultimately identifying six independent predictors: age, sex, BMI, Hb, uric acid, and creatinine (Supplementary Table S3).

### 3.3 Model development and performance comparison

The hospital dataset was randomly split into a training set (70%, *n* = 752) and an internal validation set (30%, *n* = 322) using a 7:3 ratio. Seven machine learning models were developed on the training set with 5-fold cross-validation: logistic regression, decision tree, random forest, XGBoost, LightGBM, SVM, and ANN. All models were evaluated on the validation set using AUC, calibration curves, and DCA.

**Figure 3:**
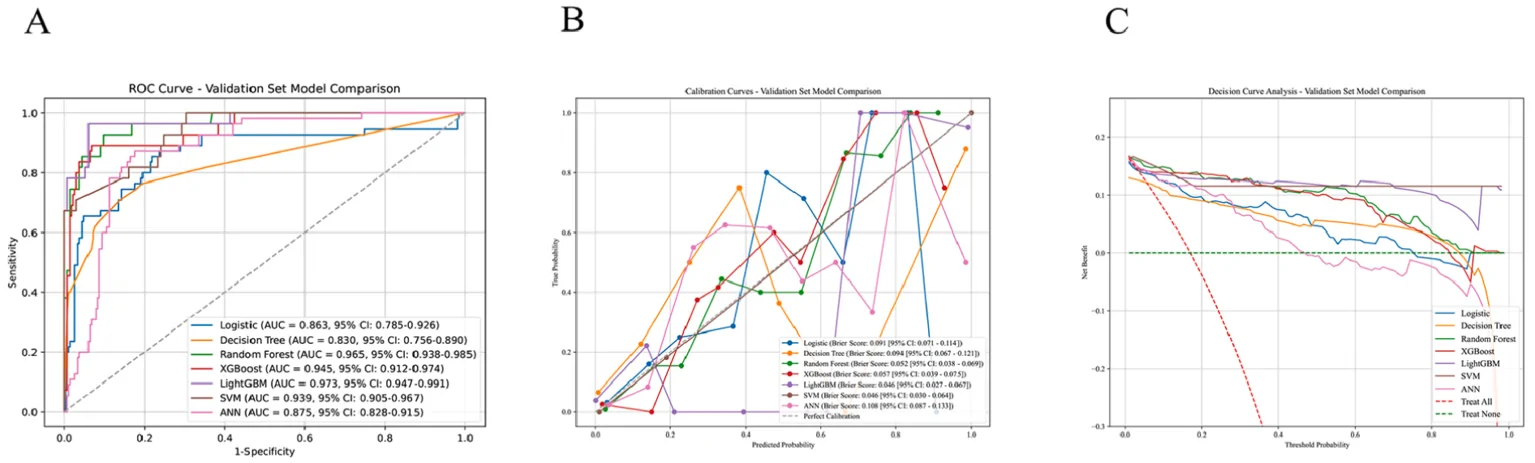
Model performance on the internal validation set. **(A)** Receiver operating characteristic (ROC) curves. **(B)** Calibration curves with Brier scores. **(C)** Decision curve analysis (DCA).

Detailed performance metrics of the models on the training set are provided in Supplementary Table S4 and Supplementary Figure S2. Performance metrics for each model on the validation set are presented in Table 2. LightGBM achieved the highest AUC (0.973, 95% CI: 0.947–0.991), followed by random forest (0.965, 95% CI: 0.938–0.985) and XGBoost (0.945, 95% CI: 0.912–0.974) (Figure 3A). LightGBM also outperformed the other models in accuracy (0.947), precision (0.896), sensitivity (0.782), specificity (0.981), and F1 score (0.835). Logistic regression had an AUC of 0.863 (95% CI: 0.785–0.926).

**Table 2:** Performance metrics for 7 models in the internal validation set.

Model	AUC	Accuracy	Precision	Sensitivity	Specificity	F1 score
Logistic	0.863	0.875	0.759	0.400	0.974	0.524
Decision Tree	0.830	0.872	0.667	0.509	0.947	0.577
Random Forest	0.965	0.935	0.854	0.745	0.974	0.796
XGBoost	0.945	0.928	0.864	0.691	0.977	0.768
LightGBM	0.973	0.947	0.896	0.782	0.981	0.835
SVM	0.939	0.944	1.000	0.673	1.000	0.804
ANN	0.875	0.822	0.471	0.291	0.932	0.360

ANN, Artificial neural network; AUC, Area under the receiver operating characteristic curve; SVM, Support vector machine.

The DeLong test showed that the AUC difference between LightGBM and random forest was not statistically significant (*p* = 0.0546). However, LightGBM significantly outperformed other models (*p* < 0.05) (Supplementary Table S5). Calibration curves were quantitatively evaluated using the Brier score: LightGBM had a Brier score of 0.046, random forest 0.052, and logistic regression 0.091, indicating that LightGBM exhibited the best probability calibration (Figure 3B). DCA further confirmed that LightGBM provided the highest net benefit across a wide range of clinically relevant threshold probabilities, followed by random forest (Figure 3C). Given its superior discriminative ability and favorable clinical utility, LightGBM was selected as the best performing model on the validation set.

### 3.4 Model explainability

**Figure 4:**
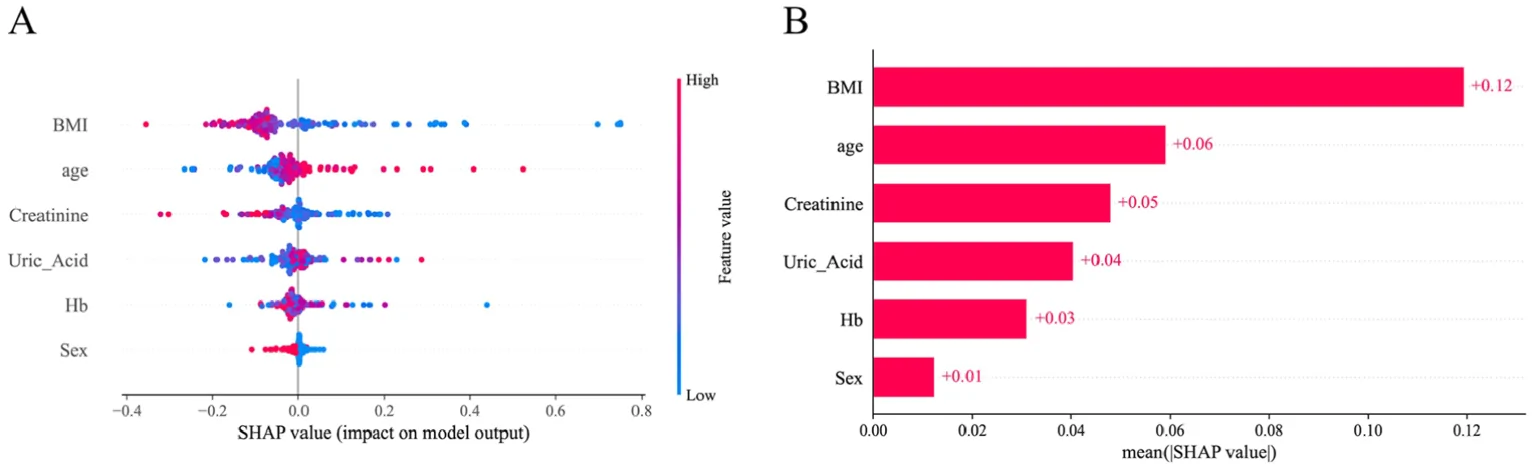
SHAP analysis of the LightGBM model: **(A)** Beeswarm plot showing the contribution of each feature to the model’s predictions. **(B)** Bar plot of mean absolute SHAP values, ranking feature importance.

The optimal LightGBM model from the validation set was selected for SHAP analysis. The SHAP beeswarm plot (Figure 4A) quantified the overall contribution of each feature to model predictions. Age and female sex served as a positive predictor for sarcopenia, whereas BMI, creatinine, uric acid, and Hb functioned as negative predictors, with lower levels corresponding to higher predicted probabilities of sarcopenia. Feature importance in this model was ranked as follows: BMI, age, creatinine, uric acid, Hb, sex (Figure 4B).

**Figure 5:**
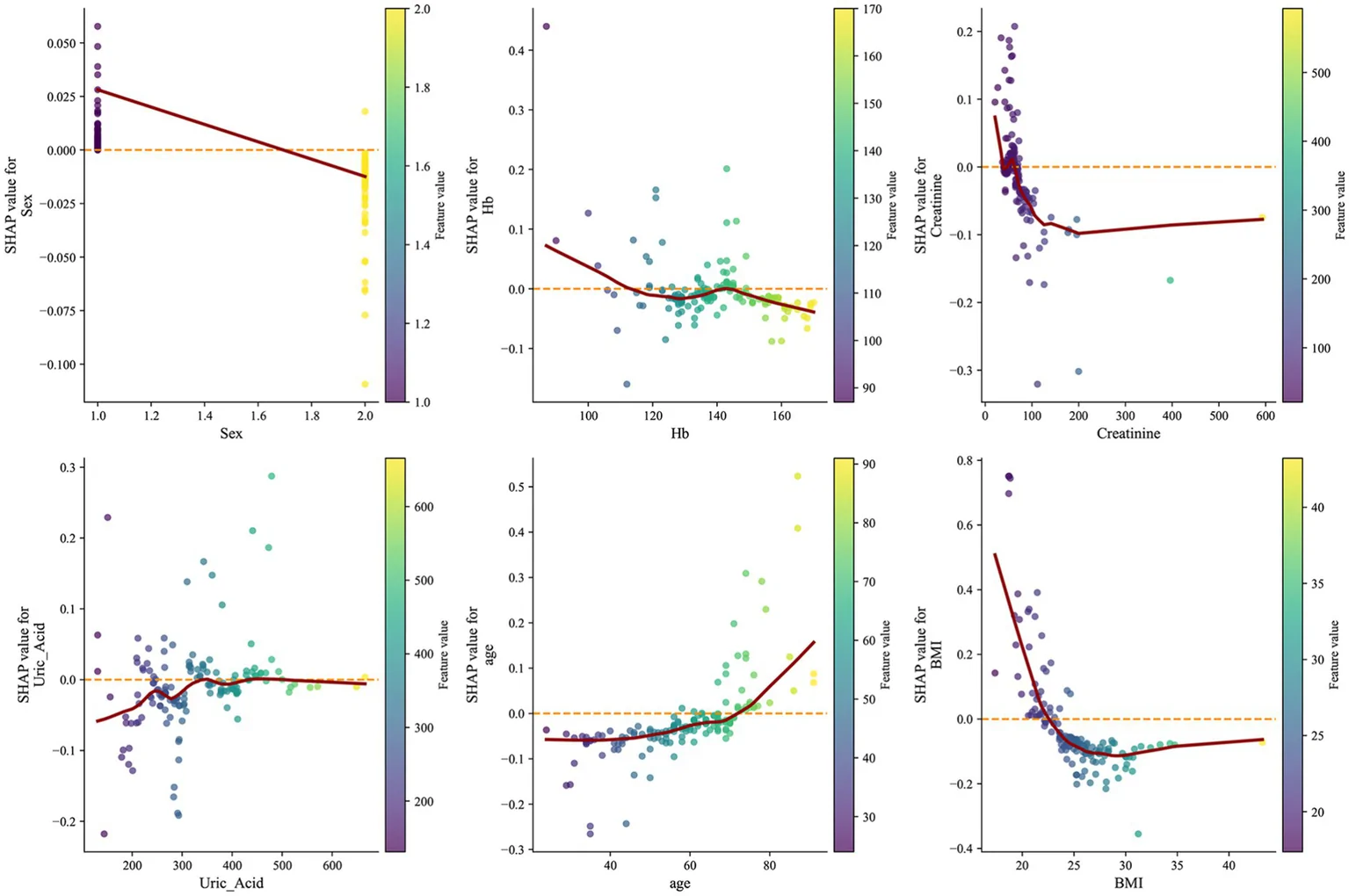
SHAP dependence plots.

SHAP dependence plots revealed nonlinear associations between each feature and sarcopenia prediction risk (Figure 5). Female sex corresponded to higher SHAP values. Hb showed a nonlinear negative association, with low Hb (<110 g/L) corresponding to markedly elevated SHAP values. Serum creatinine demonstrated a strong nonlinear negative association, with low creatinine (<100 μmol/L) corresponding to substantially elevated SHAP values. Uric acid exhibited a complex nonlinear relationship, with both extremely low and high levels (>500 μmol/L) corresponding to elevated SHAP values and moderate levels (300 to 400 μmol/L) clustering around neutrality. Age showed a monotonic positive association, with advanced age (>70 years) corresponding to significantly elevated SHAP values. BMI showed a pronounced nonlinear negative association, with low BMI (<20 kg/m^2^) corresponding to the highest SHAP values and the nadir occurring at BMI 25 to 30 kg/m^2^.

### 3.5 External validation

**Figure 6:**
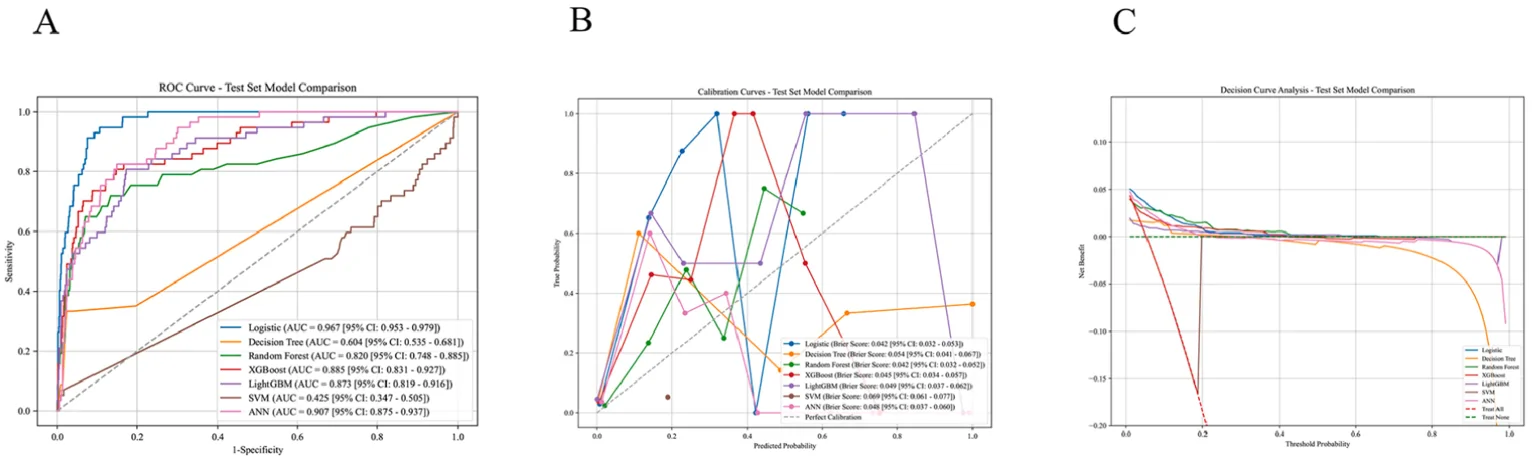
External validation using the NHANES dataset. **(A)** Receiver operating characteristic (ROC) curves. **(B)** Calibration curves with Brier scores. **(C)** Decision curve analysis (DCA).

We evaluated the performance of seven machine learning models for predicting sarcopenia risk in patients with DM using the external NHANES and CHARLS test dataset. NHANES included 1,078 patients with DM, with 1,021 non-sarcopenia (94.71%) and 57 sarcopenia (5.29%) (Supplementary Table S6). Logistic regression achieved the highest AUC (0.967, 95% CI: 0.953–0.978), with an accuracy of 0.949, precision of 1.000, specificity of 1.000, sensitivity of 0.335, and F1 score of 0.502 (Figure 6A; Supplementary Table S7). The DeLong test indicated that the AUC of the logistic regression model was significantly higher than those of the other models, with statistical significance (Supplementary Table S8). The sensitivity and F1 scores of the remaining models were all lower than those of logistic regression. The AUC of LightGBM was 0.873, with a sensitivity of 0.353 and an F1 score of 0.480. Calibration curves showed that logistic regression had the lowest Brier score of 0.042, indicating the highest agreement between predicted and observed probabilities of sarcopenia (Figure 6B). DCA demonstrated that logistic regression had certain clinical net benefit (Figure 6C).

**Figure 7:**
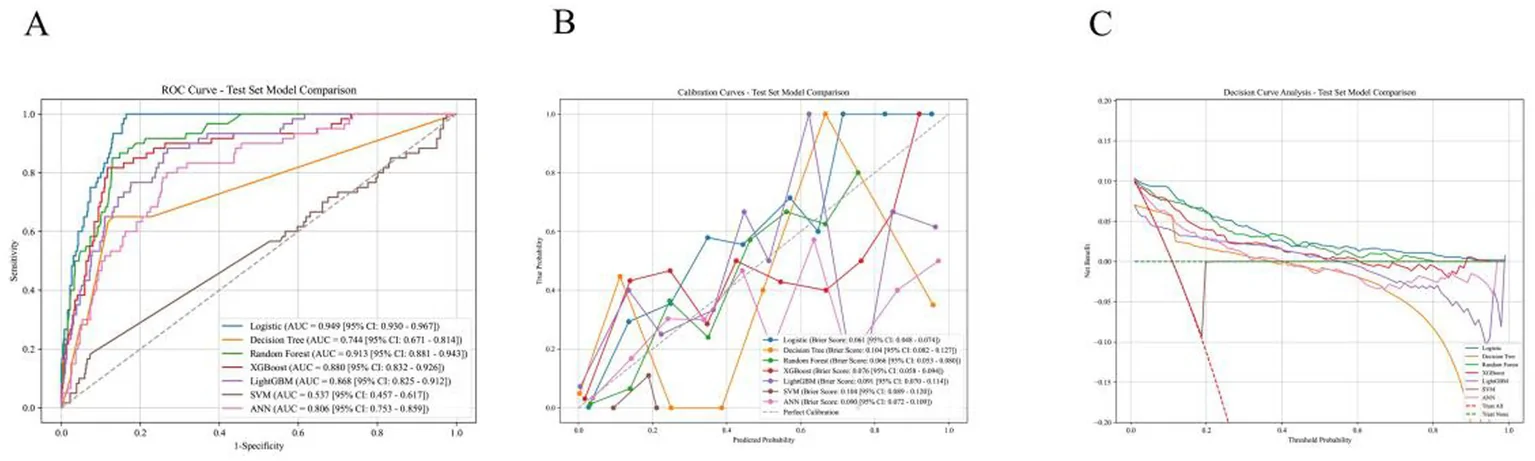
External validation using the CHARLS dataset. **(A)** Receiver operating characteristic (ROC) curves. **(B)** Calibration curves with Brier scores. **(C)** Decision curve analysis (DCA).

CHARLS included 547 patients, with 487 non-sarcopenia (89.03%) and 60 sarcopenia (10.97%) (Supplementary Table S9). In the CHARLS dataset, logistic regression again achieved the highest AUC (0.949, 95% CI: 0.930–0.967), with an accuracy of 0.910, precision of 0.789, sensitivity of 0.250, specificity of 0.992, and F1 score of 0.380 (Figure 7A; Supplementary Table S10). The DeLong test indicated that the AUC of the logistic regression model was significantly higher than those of the other models (Supplementary Table S11). Random forest had an AUC of 0.913 (95% CI: 0.881–0.943), lower than that of logistic regression, but its sensitivity (0.300) and F1 score (0.419) were slightly higher than those of logistic regression. The AUC of LightGBM was 0.868, with a sensitivity of 0.267 and an F1 score of 0.360. Calibration curves showed that logistic regression had the lowest Brier score of 0.061, indicating the highest agreement between predicted and observed probabilities of sarcopenia (Figure 7B). DCA showed that logistic regression had higher clinical net benefit than the other models (Figure 7C).

## 4 Discussion

In this study, we developed and externally validated a machine learning based predictive model for sarcopenia risk in patients with diabetes mellitus using data from a single-center retrospective cross-sectional study combined with two public external databases (NHANES and CHARLS). After systematic comparison of seven machine learning algorithms, logistic regression demonstrated superior and most stable discriminative performance in external validation, although its internal validation performance was lower than that of LightGBM. The final model included only six readily available clinical variables: age, sex, BMI, hemoglobin, uric acid, and creatinine. SHAP analysis further revealed that low BMI, older age, and low creatinine were the strongest predictors of sarcopenia risk. The model exhibited good calibration and clinical net benefit, providing a practical tool for early sarcopenia screening in diabetic patients.

In internal validation, ensemble models, particularly LightGBM and random forest, achieved comparatively higher AUCs, with values exceeding that of logistic regression. This result is within expectation, as gradient boosting tree algorithms can capture complex nonlinear interactions and possess a relatively strong fitting capacity for training data (39). However, on the two external datasets, the AUC of LightGBM decreased to 0.873 and 0.868 respectively, whereas logistic regression maintained an AUC around 0.95. This performance discrepancy may suggest that the complex models captured certain center-specific patterns from the single-center data, such as measurement biases, population composition, or clinical practice patterns. In contrast, logistic regression, with its simple structure and inherent regularization, may be less affected by single-center specificities, thereby showing relatively stable discriminative performance across different populations and different sarcopenia definitions (40). External validation is widely considered an important approach for assessing model generalizability. Therefore, although logistic regression was not the top performer internally, its stable performance across diverse datasets makes it a more reliable final model.

Age is a key factor influencing sarcopenia. The prevalence of sarcopenia increases markedly with age, reaching 12 to 75% in hospitalized patients older than 70 years (41). After age 70, the number of motor neurons declines significantly, with a loss of approximately 50% of alpha motor neurons, leading to substantial decreases in muscle strength and function (42). Age-related chronic inflammation activates the ubiquitin-proteasome system (UPS), accelerating skeletal muscle protein degradation (43). Enhanced oxidative stress impairs muscle satellite cell activity and promotes apoptosis (44). Furthermore, aging-related structural and functional abnormalities, particularly mitochondrial dysfunction, play a central role in the development of sarcopenia (45, 46). The risk of sarcopenia is higher in women than in men, which may be attributed to the decline in estrogen levels after menopause (47). Reduced estrogen not only directly inhibits skeletal muscle protein synthesis but also exacerbates anabolic resistance in the diabetic state, impairing the utilization of dietary amino acids for muscle repair and growth (48). Low BMI is significantly associated with an increased risk of sarcopenia, which differs from the epidemiological observation of sarcopenic obesity (49, 50). In the diabetic population, low body weight or low BMI often reflects malnutrition and depletion of muscle reserves (51). The SHAP plot shows that risk rises sharply when BMI < 20 kg/m^2^ and is lowest at BMI 25–30 kg/m^2^, suggesting a potential protective effect of moderate BMI. However, the prevalence of sarcopenic obesity in patients with DM can reach 27% (52), and mild to moderate weight gain may accompany higher lean mass, masking muscle catabolism (53). Therefore, both low BMI and sarcopenic obesity phenotypes should be considered when assessing sarcopenia risk.

Low Hb level is an independent risk factor for sarcopenia (54). Anemia can accelerate muscle catabolism through tissue hypoxia, suppression of insulin-like growth factor-1 (IGF-1) signaling, and upregulation of proinflammatory cytokines such as IL-6 (55, 56). In diabetic patients, chronic kidney disease and inflammatory states often coexist with anemia, further exacerbating sarcopenia risk (10). Creatinine, a biomarker of kidney function, is primarily generated by skeletal muscle cells. Under stable renal function, serum creatinine has been used to estimate muscle mass in some studies (57). Low serum uric acid concentration is associated with an increased risk of sarcopenia (58). First, uric acid possesses potent antioxidant properties, scavenging free radicals and inhibiting lipid peroxidation. Low uric acid levels may weaken the body’s antioxidant defense, exacerbate oxidative stress damage, and promote skeletal muscle protein degradation and apoptosis (59, 60). Additionally, uric acid may influence muscle metabolism by modulating NLRP3 inflammasome activity; low uric acid levels may impair this regulation, thereby aggravating chronic inflammation-mediated muscle atrophy (61).

The “black box” nature of machine learning often hinders clinical adoption. This study used the SHAP method to provide global and local explanations of the final model. These visualizations not only enhance model transparency but also help clinicians understand the risk drivers for individual patients. Primary care facilities, without specialized equipment, can perform sarcopenia risk stratification for diabetic patients, thereby enabling early identification of high-risk individuals and timely nutritional interventions and resistance training.

Two previous studies developed predictive models for sarcopenia in diabetic patients. Zou et al. constructed a logistic regression model using CHARLS data, but lacked external validation and interpretability (22). Wang et al. developed a nomogram based on single-center older T2DM patients, also without external validation (23). This study has several strengths. Firstly, we applied multiple ML algorithms and performed rigorous external validation; Furthermore, we tested the model’s generalizability in two datasets of different ethnicities and healthcare systems (NHANES and CHARLS); Lastly, we provided model interpretability using SHAP. Therefore, although logistic regression was not the top performer internally, its stable performance across diverse datasets suggests it may offer advantages in generalizability for this specific prediction task, though this observation warrants further investigation.

From a global public health perspective, this model offers a practical and scalable approach for sarcopenia risk stratification in diabetic patients across diverse healthcare settings. In low- and middle-income countries where DXA and BIA are not universally accessible, our six-variable model can be implemented using routine blood tests and anthropometric measurements, requiring minimal additional cost or specialized training. The model’s robust performance across US and Chinese populations suggests potential applicability across different ethnic groups and healthcare systems. Integration of this tool into electronic health records could facilitate automated risk screening during routine diabetic care, enabling early identification of high-risk individuals and timely implementation of preventive interventions such as nutritional optimization and resistance training. Future implementation studies are warranted to evaluate the model’s impact on clinical decision-making and patient outcomes in real-world settings.

This study has several limitations. First, it was retrospective. Although strict inclusion and exclusion criteria were applied, selection bias and information bias cannot be completely avoided. Second, sarcopenia definitions differed across the three datasets: the hospital and CHARLS adopted AWGS 2025 criteria, while NHANES used EWGSOP2 criteria, and muscle mass measurement methods varied. Although we defined outcomes according to each standard, this heterogeneity might introduce some bias, but it also tested the model’s robustness under different diagnostic frameworks. Third, the hospital data came from a single center with a modest sample size; a larger sample would provide more stable estimates. Fourth, variable selection was guided by literature and clinical experience, and we did not include potentially important factors such as dietary nutrition, physical activity, medication history or diabetes duration. Fifth, the logistic regression model exhibited low sensitivity in external validation due to class imbalance, suggesting its use as an initial screening tool rather than a standalone diagnostic instrument. Future studies should employ cost-sensitive learning or resampling techniques to address class imbalance and prospectively validate model performance in more balanced populations.

## 5 Conclusion

This study developed and externally validated a logistic regression based predictive model for sarcopenia risk in diabetic patients using six readily available variables. The model shows good discrimination and calibration, supporting its use for early clinical risk screening. Future multicenter prospective studies with large sample sizes are needed to further validate its clinical utility.

## Data Availability

The original contributions presented in the study are included in the article/Supplementary material, further inquiries can be directed to the corresponding author.
