# Supplementary material for: Development, validation, and visualization of a machine learning-based predictive model for sarcopenia risk in patients with diabetes mellitus
Source: Front Med (Lausanne). 2026 Aug 14;13:1904187. doi: 10.3389/fmed.2026.1904187 (PMC13522771; doi:10.3389/fmed.2026.1904187)
Supplement: Supplementary file 1 [file Data_Sheet_1.docx]

**Supplemental materials**

**Table S1.** Sensitivity Analysis of AUC for Models of validation set before and after multiple imputation

**Table S2.** Results of variance inflation factors (VIF).

**Table S3.** Multivariable logistic regression results.

**Table S4.** Performance metrics in the training set.

**Table S5.** DeLong test in validation set.

**Table S6.** Baseline characteristics of NHANES.

**Table S7.** Performance metrics in the NHANES set.

**Table S8.** DeLong test of NHANES set.

**Table S9.** Baseline characteristics of CHARLS.

**Table S10.** Performance metrics in the CHARLS set.

**Table S11.** DeLong test of CHARLS set.

**Figure S1.** Proportions of missing variables in the hospital dataset.

**Figure S2.** Model performance on the training set.

**Table S1.** Sensitivity Analysis of AUC for Models of validation set before and after multiple imputation

| Model of validation set | before multiple imputation | after multiple imputation | DeLong test result |
| --- | --- | --- | --- |
| Logistic | 0.843 | 0.863 | *P*>0.05 |
| Decision Tree | 0.863 | 0.830 | *P*>0.05 |
| Random Forest | 0.956 | 0.965 | *P*>0.05 |
| XGBoost | 0.947 | 0.945 | *P*>0.05 |
| LightGBM | 0.962 | 0.973 | *P*>0.05 |
| SVM | 0.930 | 0.939 | *P*>0.05 |
| ANN | 0.844 | 0.875 | *P*>0.05 |

**Table S2.** Results of variance inflation factors (VIF).

| Variables |  |
| --- | --- |
| MCHC | 123.895 |
| MCV | 97.229 |
| TG | 6.659 |
| Hb | 6.246 |
| RBC | 5.549 |
| BMI | 4.355 |
| HDL.C | 3.719 |
| age | 1.955 |
| NEU | 1.874 |
| HbA1c | 1.788 |
| Creatinine | 1.550 |
| CVD | 1.207 |
| PDW | 1.176 |

**Table S3.** Multivariable logistic regression results.

| Variable | Beta | SE | Wald | OR | Lower CI | Upper CI | *P*-value |
| --- | --- | --- | --- | --- | --- | --- | --- |
| Sex | -0.65 | 0.301 | -2.16 | 0.522 | 0.29 | 0.942 | 0.031 |
| Hb | -0.023 | 0.009 | -2.543 | 0.977 | 0.96 | 0.995 | 0.011 |
| Creatinine | -0.035 | 0.008 | -4.586 | 0.965 | 0.951 | 0.98 | <0.001 |
| Uric Acid | 0.003 | 0.002 | 2.171 | 1.003 | 1 | 1.006 | 0.03 |
| age | 0.053 | 0.011 | 4.77 | 1.054 | 1.032 | 1.078 | <0.001 |
| BMI | -0.503 | 0.082 | -6.147 | 0.605 | 0.515 | 0.71 | <0.001 |

**Table S4.** Performance metrics in the training set.

| Model | AUC | Accuracy | Precision | Sensitivity | Specificity | F1 Score |
| --- | --- | --- | --- | --- | --- | --- |
| Logistic | 0.881 | 0.870 | 0.681 | 0.473 | 0.953 | 0.559 |
| Decision Tree | 0.989 | 0.959 | 0.903 | 0.855 | 0.981 | 0.878 |
| Random Forest | 1.000 | 1.000 | 1.000 | 1.000 | 1.000 | 1.000 |
| XGBoost | 1.000 | 0.992 | 0.992 | 0.962 | 0.998 | 0.977 |
| LightGBM | 1.000 | 1.000 | 1.000 | 1.000 | 1.000 | 1.000 |
| SVM | 1.000 | 1.000 | 1.000 | 1.000 | 1.000 | 1.000 |
| ANN | 0.870 | 0.863 | 0.667 | 0.427 | 0.955 | 0.521 |

**Table S5.** DeLong test in validation set.

| Model 1 | Model 2 | AUC 1 | 95% CI 1 | AUC 2 | 95% CI 2 | P value | Significant |
| --- | --- | --- | --- | --- | --- | --- | --- |
| Logistic | Decision Tree | 0.863 | [0.785, 0.926] | 0.83 | [0.756, 0.890] | 0.583 | No |
| Logistic | Random Forest | 0.863 | [0.785, 0.926] | 0.965 | [0.938, 0.985] | 0.0028 | Yes |
| Logistic | XGBoost | 0.863 | [0.785, 0.926] | 0.945 | [0.912, 0.974] | 0.0189 | Yes |
| Logistic | LightGBM | 0.863 | [0.785, 0.926] | 0.973 | [0.947, 0.991] | 0.0016 | Yes |
| Logistic | SVM | 0.863 | [0.785, 0.926] | 0.939 | [0.905, 0.967] | 0.0539 | No |
| Logistic | ANN | 0.863 | [0.785, 0.926] | 0.875 | [0.828, 0.915] | 0.7249 | No |
| Decision Tree | Random Forest | 0.83 | [0.756, 0.890] | 0.965 | [0.938, 0.985] | 0.0039 | Yes |
| Decision Tree | XGBoost | 0.83 | [0.756, 0.890] | 0.945 | [0.912, 0.974] | 0.0083 | Yes |
| Decision Tree | LightGBM | 0.83 | [0.756, 0.890] | 0.973 | [0.947, 0.991] | 0.0032 | Yes |
| Decision Tree | SVM | 0.83 | [0.756, 0.890] | 0.939 | [0.905, 0.967] | 0.0153 | Yes |
| Decision Tree | ANN | 0.83 | [0.756, 0.890] | 0.875 | [0.828, 0.915] | 0.3511 | No |
| Random Forest | XGBoost | 0.965 | [0.938, 0.985] | 0.945 | [0.912, 0.974] | 0.0461 | Yes |
| Random Forest | LightGBM | 0.965 | [0.938, 0.985] | 0.973 | [0.947, 0.991] | 0.0546 | No |
| Random Forest | SVM | 0.965 | [0.938, 0.985] | 0.939 | [0.905, 0.967] | 0.1095 | No |
| Random Forest | ANN | 0.965 | [0.938, 0.985] | 0.875 | [0.828, 0.915] | 0.0001 | Yes |
| XGBoost | LightGBM | 0.945 | [0.912, 0.974] | 0.973 | [0.947, 0.991] | 0.0175 | Yes |
| XGBoost | SVM | 0.945 | [0.912, 0.974] | 0.939 | [0.905, 0.967] | 0.7408 | No |
| XGBoost | ANN | 0.945 | [0.912, 0.974] | 0.875 | [0.828, 0.915] | 0.0035 | Yes |
| LightGBM | SVM | 0.973 | [0.947, 0.991] | 0.939 | [0.905, 0.967] | 0.0319 | Yes |
| LightGBM | ANN | 0.973 | [0.947, 0.991] | 0.875 | [0.828, 0.915] | 0.0001 | Yes |
| SVM | ANN | 0.939 | [0.905, 0.967] | 0.875 | [0.828, 0.915] | 0.0218 | Yes |

**Table S6.** Baseline characteristics of NHANES.

| Variables | Total (n = 1078) | No sarcopenia (n = 1021) | Sarcopenia (n = 57) | *P* |
| --- | --- | --- | --- | --- |
|  |  |  |  |  |
| BMI, Median (IQR) | 32.10 (28.00, 37.40) | 32.70 (28.70, 37.80) | 22.40 (21.00, 23.80) | <.001 |
| WC, Median (IQR) | 107.60 (97.43, 120.10) | 109.10 (99.20, 120.80) | 86.60 (79.50, 92.30) | <.001 |
| HDL C, Median (IQR) | 1.14 (0.96, 1.34) | 1.14 (0.96, 1.34) | 1.32 (1.09, 1.71) | <.001 |
| TC, Median (IQR) | 4.94 (4.19, 5.73) | 4.94 (4.19, 5.72) | 4.81 (4.14, 6.00) | 0.792 |
| HbA1c, Median (IQR) | 6.80 (6.10, 8.60) | 6.80 (6.10, 8.60) | 6.90 (5.70, 8.80) | 0.621 |
| Uric Acid, Median (IQR) | 321.20 (267.70, 380.70) | 327.10 (273.60, 380.70) | 291.50 (237.90, 392.60) | 0.133 |
| Creatinine, Median (IQR) | 70.72 (59.23, 83.98) | 70.72 (59.23, 84.86) | 66.30 (57.46, 74.26) | 0.016 |
| WBC, Median (IQR) | 7.60 (6.30, 9.10) | 7.70 (6.40, 9.10) | 6.90 (5.70, 8.40) | 0.011 |
| NEU, Median (IQR) | 4.50 (3.50, 5.60) | 4.50 (3.50, 5.60) | 4.20 (3.20, 5.50) | 0.196 |
| LYM, Median (IQR) | 2.30 (1.80, 2.80) | 2.30 (1.80, 2.90) | 1.90 (1.60, 2.60) | <.001 |
| MONO, Median (IQR) | 0.55 (0.40, 0.70) | 0.60 (0.40, 0.70) | 0.50 (0.40, 0.60) | 0.062 |
| RBC, Median (IQR) | 4.79 (4.47, 5.13) | 4.79 (4.47, 5.14) | 4.71 (4.40, 5.07) | 0.262 |
| Hb, Median (IQR) | 142.00 (131.00, 152.00) | 142.00 (131.00, 152.00) | 140.00 (130.00, 151.00) | 0.686 |
| MCV, Median (IQR) | 87.75 (84.00, 90.90) | 87.70 (84.00, 90.70) | 89.90 (84.50, 93.20) | 0.023 |
| MCH, Median (IQR) | 29.60 (28.10, 30.90) | 29.60 (28.00, 30.90) | 29.70 (28.50, 31.10) | 0.231 |
| MCHC, Median (IQR) | 336.00 (329.00, 343.00) | 336.00 (329.00, 343.00) | 336.00 (328.00, 340.00) | 0.274 |
| RDW, Median (IQR) | 13.50 (12.90, 14.30) | 13.50 (13.00, 14.30) | 13.30 (12.80, 14.00) | 0.029 |
| PLT, Median (IQR) | 241.00 (203.00, 291.00) | 241.00 (203.00, 291.00) | 247.00 (203.00, 291.00) | 0.910 |
| Age, Median (IQR) | 49.00 (42.00, 54.00) | 49.00 (42.00, 54.00) | 51.00 (39.00, 55.00) | 0.594 |
| Sex, n (%) |  |  |  | 0.215 |
| Male | 540 (50.09) | 516 (50.54) | 24 (42.11) |  |
| Female | 538 (49.91) | 505 (49.46) | 33 (57.89) |  |
| Smoking, n (%) |  |  |  | 0.561 |
| No | 622 (57.70) | 587 (57.49) | 35 (61.40) |  |
| Yes | 456 (42.30) | 434 (42.51) | 22 (38.60) |  |
| Alcohol, n (%) |  |  |  | 0.160 |
| No | 174 (16.14) | 161 (15.77) | 13 (22.81) |  |
| Yes | 904 (83.86) | 860 (84.23) | 44 (77.19) |  |
| Hypertension, n (%) |  |  |  | 0.005 |
| No | 486 (45.08) | 450 (44.07) | 36 (63.16) |  |
| Yes | 592 (54.92) | 571 (55.93) | 21 (36.84) |  |
| Stroke, n (%) |  |  |  | 0.760 |
| No | 1042 (96.66) | 986 (96.57) | 56 (98.25) |  |
| Yes | 36 (3.34) | 35 (3.43) | 1 (1.75) |  |
| CVD, n (%) |  |  |  | 0.266 |
| No | 976 (90.54) | 922 (90.30) | 54 (94.74) |  |
| Yes | 102 (9.46) | 99 (9.70) | 3 (5.26) |  |

**Table S7.** Performance metrics in the NHANES set.

| Model | AUC | Accuracy | Precision | Sensitivity | Specificity | F1 Score |
| --- | --- | --- | --- | --- | --- | --- |
| Logistic | 0.967 | 0.949 | 1.000 | 0.335 | 1.000 | 0.502 |
| Decision Tree | 0.604 | 0.939 | 0.263 | 0.388 | 0.986 | 0.314 |
| Random Forest | 0.820 | 0.948 | 0.667 | 0.335 | 0.999 | 0.446 |
| XGBoost | 0.885 | 0.946 | 0.333 | 0.318 | 0.998 | 0.325 |
| LightGBM | 0.873 | 0.949 | 0.750 | 0.353 | 0.999 | 0.480 |
| SVM | 0.425 | 0.947 | 0.000 | 0.300 | 1.000 | 0.000 |
| ANN | 0.907 | 0.943 | 0.000 | 0.300 | 0.996 | 0.000 |

**Table S8.** DeLong test of NHANES set.

| Model 1 | Model 2 | AUC 1 | 95% CI 1 | AUC 2 | 95% CI 2 | P value | Significant |
| --- | --- | --- | --- | --- | --- | --- | --- |
| Logistic | Decision Tree | 0.967 | [0.953, 0.979] | 0.604 | [0.535, 0.681] | 0 | Yes |
| Logistic | Random Forest | 0.967 | [0.953, 0.979] | 0.82 | [0.748, 0.885] | 0 | Yes |
| Logistic | XGBoost | 0.967 | [0.953, 0.979] | 0.885 | [0.831, 0.927] | 0.0001 | Yes |
| Logistic | LightGBM | 0.967 | [0.953, 0.979] | 0.873 | [0.819, 0.916] | 0 | Yes |
| Logistic | SVM | 0.967 | [0.953, 0.979] | 0.425 | [0.347, 0.505] | 0 | Yes |
| Logistic | ANN | 0.967 | [0.953, 0.979] | 0.907 | [0.875, 0.937] | 0 | Yes |
| Decision Tree | Random Forest | 0.604 | [0.535, 0.681] | 0.82 | [0.748, 0.885] | 0.0003 | Yes |
| Decision Tree | XGBoost | 0.604 | [0.535, 0.681] | 0.885 | [0.831, 0.927] | 0 | Yes |
| Decision Tree | LightGBM | 0.604 | [0.535, 0.681] | 0.873 | [0.819, 0.916] | 0 | Yes |
| Decision Tree | SVM | 0.604 | [0.535, 0.681] | 0.425 | [0.347, 0.505] | 0.0152 | Yes |
| Decision Tree | ANN | 0.604 | [0.535, 0.681] | 0.907 | [0.875, 0.937] | 0 | Yes |
| Random Forest | XGBoost | 0.82 | [0.748, 0.885] | 0.885 | [0.831, 0.927] | 0.0167 | Yes |
| Random Forest | LightGBM | 0.82 | [0.748, 0.885] | 0.873 | [0.819, 0.916] | 0.0251 | Yes |
| Random Forest | SVM | 0.82 | [0.748, 0.885] | 0.425 | [0.347, 0.505] | 0 | Yes |
| Random Forest | ANN | 0.82 | [0.748, 0.885] | 0.907 | [0.875, 0.937] | 0.0276 | Yes |
| XGBoost | LightGBM | 0.885 | [0.831, 0.927] | 0.873 | [0.819, 0.916] | 0.3932 | No |
| XGBoost | SVM | 0.885 | [0.831, 0.927] | 0.425 | [0.347, 0.505] | 0 | Yes |
| XGBoost | ANN | 0.885 | [0.831, 0.927] | 0.907 | [0.875, 0.937] | 0.3809 | No |
| LightGBM | SVM | 0.873 | [0.819, 0.916] | 0.425 | [0.347, 0.505] | 0 | Yes |
| LightGBM | ANN | 0.873 | [0.819, 0.916] | 0.907 | [0.875, 0.937] | 0.1906 | No |
| SVM | ANN | 0.425 | [0.347, 0.505] | 0.907 | [0.875, 0.937] | 0 | Yes |

**Table S9.** Baseline characteristics of CHARLS.

| Variables | Total (n = 547) | No sarcopenia (n = 487) | Sarcopenia (n = 60) | *P* |
| --- | --- | --- | --- | --- |
|  |  |  |  |  |
| WBC, Median (IQR) | 6.29 (5.20, 7.60) | 6.29 (5.20, 7.70) | 6.20 (5.00, 7.40) | 0.383 |
| MCV, Median (IQR) | 89.90 (85.85, 93.95) | 89.90 (86.00, 93.90) | 89.35 (85.47, 94.25) | 0.798 |
| HDL C, Median (IQR) | 1.13 (0.94, 1.34) | 1.10 (0.92, 1.32) | 1.30 (1.13, 1.50) | <.001 |
| LDL C, Median (IQR) | 3.10 (2.48, 3.74) | 3.07 (2.48, 3.73) | 3.17 (2.50, 3.77) | 0.717 |
| TC, Median (IQR) | 5.09 (4.47, 5.77) | 5.12 (4.48, 5.76) | 4.95 (4.38, 5.93) | 0.938 |
| TG, Median (IQR) | 1.49 (1.03, 2.29) | 1.53 (1.06, 2.38) | 1.16 (0.86, 1.66) | <.001 |
| FBG, Median (IQR) | 7.86 (6.18, 10.74) | 7.93 (6.22, 10.71) | 7.23 (6.04, 13.03) | 0.624 |
| Creatinine, Median (IQR) | 65.93 (57.94, 78.91) | 66.93 (57.94, 79.41) | 63.93 (53.69, 75.92) | 0.060 |
| Uric Acid, Median (IQR) | 251.80 (206.07, 311.68) | 253.90 (208.22, 315.57) | 225.31 (184.48, 270.39) | 0.001 |
| HbA1c, Median (IQR) | 6.10 (5.30, 7.60) | 6.10 (5.40, 7.55) | 5.80 (5.17, 8.22) | 0.676 |
| Age, Median (IQR) | 60.00 (54.00, 66.00) | 59.00 (54.00, 65.00) | 64.50 (60.00, 70.00) | <.001 |
| BMI, Median (IQR) | 25.43 (23.20, 27.91) | 26.01 (23.84, 28.25) | 20.53 (18.69, 21.71) | <.001 |
| WC, Median (IQR) | 92.00 (85.00, 98.20) | 93.00 (87.00, 99.20) | 81.50 (74.25, 85.00) | <.001 |
| Hb, Median (IQR) | 142.00 (130.00, 155.00) | 143.00 (130.00, 156.00) | 136.00 (127.00, 144.25) | 0.004 |
| Sex, n (%) |  |  |  | <.001 |
| Male | 222 (40.59) | 210 (43.12) | 12 (20.00) |  |
| Female | 325 (59.41) | 277 (56.88) | 48 (80.00) |  |
| Hypertension, n (%) |  |  |  | <.001 |
| No | 252 (46.07) | 210 (43.12) | 42 (70.00) |  |
| Yes | 295 (53.93) | 277 (56.88) | 18 (30.00) |  |
| CVD, n (%) |  |  |  | 0.387 |
| No | 408 (74.59) | 366 (75.15) | 42 (70.00) |  |
| Yes | 139 (25.41) | 121 (24.85) | 18 (30.00) |  |
| Stroke, n (%) |  |  |  | 0.613 |
| No | 514 (93.97) | 459 (94.25) | 55 (91.67) |  |
| Yes | 33 (6.03) | 28 (5.75) | 5 (8.33) |  |
| Smoking, n (%) |  |  |  | 0.014 |
| No | 370 (67.64) | 321 (65.91) | 49 (81.67) |  |
| Yes | 177 (32.36) | 166 (34.09) | 11 (18.33) |  |
| Alcohol, n (%) |  |  |  | 0.055 |
| No | 420 (76.78) | 368 (75.56) | 52 (86.67) |  |
| Yes | 127 (23.22) | 119 (24.44) | 8 (13.33) |  |

**Table S10.** Performance metrics in the CHARLS set.

| Model | AUC | Accuracy | Precision | Sensitivity | Specificity | F1 Score |
| --- | --- | --- | --- | --- | --- | --- |
| Logistic | 0.949 | 0.910 | 0.789 | 0.250 | 0.992 | 0.380 |
| Decision Tree | 0.744 | 0.878 | 0.415 | 0.283 | 0.951 | 0.337 |
| Random Forest | 0.913 | 0.909 | 0.692 | 0.300 | 0.984 | 0.419 |
| XGBoost | 0.880 | 0.898 | 0.567 | 0.283 | 0.973 | 0.378 |
| LightGBM | 0.868 | 0.896 | 0.552 | 0.267 | 0.973 | 0.360 |
| SVM | 0.537 | 0.890 | 0.000 | 0.000 | 1.000 | 0.000 |
| ANN | 0.806 | 0.878 | 0.360 | 0.150 | 0.967 | 0.212 |

**Table S11.** DeLong test of CHARLS set.

| Model 1 | Model 2 | AUC 1 | 95% CI 1 | AUC 2 | 95% CI 2 | P value | Significant |
| --- | --- | --- | --- | --- | --- | --- | --- |
| Logistic | Decision Tree | 0.949 | [0.930, 0.967] | 0.744 | [0.671, 0.814] | 0.0004 | Yes |
| Logistic | Random Forest | 0.949 | [0.930, 0.967] | 0.913 | [0.881, 0.943] | 0.0024 | Yes |
| Logistic | XGBoost | 0.949 | [0.930, 0.967] | 0.88 | [0.832, 0.926] | 0.0008 | Yes |
| Logistic | LightGBM | 0.949 | [0.930, 0.967] | 0.868 | [0.825, 0.912] | 0 | Yes |
| Logistic | SVM | 0.949 | [0.930, 0.967] | 0.537 | [0.457, 0.617] | 0 | Yes |
| Logistic | ANN | 0.949 | [0.930, 0.967] | 0.806 | [0.753, 0.859] | 0 | Yes |
| Decision Tree | Random Forest | 0.744 | [0.671, 0.814] | 0.913 | [0.881, 0.943] | 0.002 | Yes |
| Decision Tree | XGBoost | 0.744 | [0.671, 0.814] | 0.88 | [0.832, 0.926] | 0.008 | Yes |
| Decision Tree | LightGBM | 0.744 | [0.671, 0.814] | 0.868 | [0.825, 0.912] | 0.0239 | Yes |
| Decision Tree | SVM | 0.744 | [0.671, 0.814] | 0.537 | [0.457, 0.617] | 0.0016 | Yes |
| Decision Tree | ANN | 0.744 | [0.671, 0.814] | 0.806 | [0.753, 0.859] | 0.3162 | No |
| Random Forest | XGBoost | 0.913 | [0.881, 0.943] | 0.88 | [0.832, 0.926] | 0.0138 | Yes |
| Random Forest | LightGBM | 0.913 | [0.881, 0.943] | 0.868 | [0.825, 0.912] | 0 | Yes |
| Random Forest | SVM | 0.913 | [0.881, 0.943] | 0.537 | [0.457, 0.617] | 0 | Yes |
| Random Forest | ANN | 0.913 | [0.881, 0.943] | 0.806 | [0.753, 0.859] | 0.0002 | Yes |
| XGBoost | LightGBM | 0.88 | [0.832, 0.926] | 0.868 | [0.825, 0.912] | 0.3596 | No |
| XGBoost | SVM | 0.88 | [0.832, 0.926] | 0.537 | [0.457, 0.617] | 0 | Yes |
| XGBoost | ANN | 0.88 | [0.832, 0.926] | 0.806 | [0.753, 0.859] | 0.0332 | Yes |
| LightGBM | SVM | 0.868 | [0.825, 0.912] | 0.537 | [0.457, 0.617] | 0 | Yes |
| LightGBM | ANN | 0.868 | [0.825, 0.912] | 0.806 | [0.753, 0.859] | 0.0401 | Yes |
| SVM | ANN | 0.537 | [0.457, 0.617] | 0.806 | [0.753, 0.859] | 0 | Yes |

**Figure S1.** Proportions of missing variables in the hospital dataset.

**Figure S2.** Model performance on the training set.
